# Epitranscriptomic profile of *Lactobacillus agilis* and its adaptation to growth on inulin

**DOI:** 10.1186/s13104-021-05563-2

**Published:** 2021-04-21

**Authors:** Hongzhou Wang, Jennifer H. Simpson, Madison E. Kotra, Yuanting Zhu, Saumya Wickramasinghe, David A. Mills, Norman H. L. Chiu

**Affiliations:** 1 Department of Chemistry and Biochemistry, University of North Carolina Greensboro, Greensboro, NC USA; 2 Department of Food Science and Technology, University of California, Davis, CA USA; 3 Joint School of Nanoscience and Nanoengineering, University of North Carolina Greensboro, Greensboro, NC USA

**Keywords:** RNA modifications, Epitranscriptome, *Lactobacillus agilis*, Inulin

## Abstract

**Objective:**

Ribonucleic acids (RNA) are involved in many cellular functions. In general, RNA is made up by only four different ribonucleotides. The modifications of RNA (epitranscriptome) can greatly enhance the structural diversity of RNA, which in turn support some of the RNA functions. To determine whether the epitranscriptome of a specific probiotic is associated with its adaptation to the source of energy, *Lactobacillus agilis* (YZ050) was selected as a model and its epitranscriptome was profiled and compared by using mass spectrometry.

**Results:**

The *L. agilis* epitranscriptome (minus rRNA modifications) consists of 17 different RNA modifications. By capturing the *L. agilis* cells during exponential growth, reproducible profiling was achieved. In a comparative study, the standard source of energy (glucose) in the medium was substituted by a prebiotic inulin, and a downward trend in the *L. agilis* epitranscriptome was detected. This marks the first report on a system-wide variation of a bacterial epitranscriptome that resulted from adapting to an alternative energy source. No correlation was found between the down-regulated RNA modifications and the expression level of corresponding writer genes. Whereas, the expression level of a specific exonuclease gene, RNase J1, was detected to be higher in cells grown on inulin.

## Introduction

Collectively, all the RNA molecules in a specific group of cells are referred as a transcriptome. In order to achieve some of the RNA functionalities, the RNA structure can be altered by more than 170 different RNA modifications [1, 2]. The presence of a RNA modification is the result of an enzymatic reaction of its corresponding writer enzyme. In contrast, RNA modification can be removed by a different enzyme called eraser. To recognize the importance of RNA modifications to the RNA structures and functions, the term of epitranscriptome was coined by Mason and his associates [3]. There are reports indicating specific epitranscriptomes are linked to a variety of health-related issues [4, 5]. With the interests in studying epitranscriptomes, a number of methods for analyzing RNA modifications have been developed [6, 7]. Among those methods, mass spectrometric (MS) based method is the only universal approach for detecting different RNA modifications.

*Lactobacillus* species are common constituents of gastrointestinal tracts [8], and have been used as probiotics [9]. Prebiotics are defined as substrates that are utilized by microorganisms conferring health benefits [10]. One of the most commonly used prebiotics is inulin [11]. Since inulin cannot be metabolized by human digestive enzymes, the digestion of inulin relies on gut microbes [12]. In this report, we use the MS method to profile the *L. agilis* epitranscriptome, and subsequently determine whether the *L. agilis* epitranscriptome is involved in the adaptation to inulin.

## Main text

### Methods

*E. coli* alkaline phosphatase, Benzonase nuclease and bovine serum albumin (BSA) were purchased from Sigma-Aldrich (St. Louis, MO, USA). The venom exonuclease phosphodiesterase I was purchased from Worthington Biochemical Corp. (Lakewood, NJ, USA). All other solvents were purchased from Thermo Fisher Scientific (Waltham, MA).

#### Culturing of *L. agilis*

A *Lactobacillus agilis* strain named YZ050 was previously isolated from dairy cow fecal samples in our lab and showed the capability to ferment inulin [13]. The stock was streaked on MRS plates. After 24 h, a MRS broth was inoculated and cultivated at 37 °C under anaerobic conditions. The overnight culture (1%) was inoculated into basal MRS media supplemented with 1% inulin or 1% glucose. After ~ 5 h, samples were taken for RNA extraction at mid-log phase.

#### Extraction of RNA

Cells were pelleted and resuspended in 1 mL RNAprotect Bacteria Reagent (Qiagen Inc, Valencia). The cells were washed twice with 1X PBS and pre-lysed with 250 μL 50 g/L lysozyme and 120 μL 1000 units/mL mutanolysin. Total RNA was extracted using the RNeasy mini kit (Qiagen Inc, Valencia). Total RNA samples were DNase-treated twice, and the absence of genomic DNA was confirmed by PCR.

#### Depletion of ribosomal RNA (rRNA)

Ribosomal RNA was removed using the RiboMinus Transcriptome Isolation Kit, (Thermo Fisher, Waltham, MA). The integrity of RNA was assayed using the 2100 Bioanalyzer (Agilent Technologies, Santa Clara, CA).

#### Digestion of rRNA-depleted RNA

Each RNA sample was digested in an enzymatic reaction of 25 µL at 37 °C for 3 h, which contained 5 µg rRNA-depleted RNA, 0.05 units phosphodiesterase I, 0.5 units alkaline phosphatase, 5 units benzonase, 50 mM Tris–HCl (pH 8.0), 1 mM MgCl_2_ and 0.1 mg/mL BSA [14]. After removing the enzymes with 3 K MWCO spin filter at 14,000*g* for 15 min (Pall Corporation, Port Washington, NY), the digested RNA sample was diluted in deionized water to 50 ng/µL.

#### UPLC-MS/MS analysis of digested RNA

An Acquity ultra-high performance liquid chromatography (UPLC) system (Waters Corporation, Milford, MA) which was equipped with an Acquity HSS T3 column (2.1 × 50 mm, 1.8 µm) and a HSS T3 VanGuard pre-column (2.1 × 5 mm, 1.8 µm) at 30 °C was used. After injecting 10 µL of sample, the elution was carried out with a binary solvent system, in which solvent A contained water and 0.01% (v/v) formic acid, and solvent B contained 50% acetonitrile and 0.01% (v/v) formic acid at a flowrate of 0.4 mL/min. The gradient elution profile initiated at 100:0 (A:B) from 0.0 to 0.5 min., ramping to 70:30 from 0.5 to 9 min, followed by 50:50 from 9 to 10 min, and ended with 0:100 from 10 to 17 min. Randomized injections were used. The negative control was prepared without any RNA sample.

Tandem mass spectrometry (MS/MS) was performed on a Q Exactive Plus (Thermo Fisher Scientific, Waltham, MA) in the positive mode with ESI at 425 °C and 3.5 kV. Sheath and auxiliary gas flow were at 50 and 13 arbitrary units, respectively. Data was acquired with an inclusion list of calculated m/z of all known RNA modifications. The mass calibration was performed using a canonical ribonucleoside standard mixture (3 ng/µL). Data analysis was carried out with Xcalibur (Thermo Fisher Scientific, Waltham, MA) restricting the precursor ion to ≤ 5 ppm accuracy and its retention time to ≤ 0.1 min.

#### RNA sequencing (RNAseq)

Each library was generated from 20 ng rRNA-depleted RNA sample using the Kapa Hyper Stranded RNA-seq kit (KapaBiosystems, Cape Town, South Africa). The consistency of the libraries was verified by 2100 Bioanalyzer. The libraries were quantified by fluorometry and sequenced on a NextSeq 500 (Illumina, San Diego, CA) with paired-end 75p reads. The sequence files were processed using the CLC-Bio Genomics Workbench (CLC Bio, Denmark).

## Results and discussion

### Analysis of *L. agilis* epitranscriptome

The notions for epitranscriptome to be a standalone investigation include a single RNA modification can potentially alter the RNA interactions [15]. There are also evidence showing unique epitranscriptomes are associated with specific phenotypes [16]. Together with the discovery of various writer genes for RNA modifications, a specific epitranscriptome is considered to represent a set of specific codes for regulating cellular activities [17]. Our initial efforts focused on establishing the profile of *L. agilis* epitranscriptome. Among various types of RNA, ribosomal RNA (rRNA) makes up ~ 80% of total RNA [18]. To better witness bacterial gene expression, rRNA is often depleted from the RNA samples prior to sequencing. Equivalently, rRNA was also removed in our protocol, otherwise would reduce the detectability of RNA modifications that are unique in other types of RNA. The removal of rRNA can also enhance our ability to detect any variations on the levels of some specific RNA modifications.

The results obtained from analyzing all the detectable ribonucleosides in a *L. agilis* sample with a signal-to-noise ratio of ≥ 2 are shown in Table 1. To ensure the low abundant RNA modifications could be detected, the chromatography and signal intensity in the UPLC-MS/MS analysis were optimized. As low as 0.4 pg/μL of each canonical ribonucleoside standard were detected in our calibration experiments. For identifying the RNA modification, both MS and MS/MS data must match with the expected values with < 5 ppm error. For the MS/MS measurements, at least two fragment ions were identifiable. The profiling was repeated four times with different samples, and the same profile of RNA modifications were detected each time. To the best of our knowledge, this is the first time the profile of *L. agilis* epitranscriptome (minus the rRNA modifications) is reported.

**Table 1:** LC–MS data obtained from the glucose-associated *L. agilis* transcriptome in the absence of rRNA

Ribonucleoside detected and its short name	Retention time^a^ (min)	Measured mass^b^ (Da)	Mass accuracy^c^ (ppm)
Cytidine, C	0.90	244.0935	0.7
Dihydrouridine, D	0.94	247.0933	3.3
Pseudouridine, Y	0.98	245.0775	3.0
1-Methyladenosine, m1A	1.55	282.1205	3.1
5-Methylcytidine, m5C	1.63	258.1093	3.2
Uridine, U	2.00	245.0776	3.3
7-Methylguanosine, m7G	2.54	298.1155	3.1
2′-O-Methylcytidine, Cm	2.79	258.1093	3.2
2′-O-Methylpseudouridine, Ym	2.99	259.0934	3.5
Guanosine, G	3.99	284.0997	2.8
5-Methyluridine, m5U	4.06	259.0933	3.2
Adenosine, A	4.45	268.1048	2.8
3-Methyluridine, m3U	4.86	259.0933	3.1
1-Methylguanosine, m1G	5.27	298.1155	3.2
2′-O-Methylguanosine, Gm	5.27	298.1155	3.2
N2-methylguanosine, m2G	5.53	298.1155	3.2
N4-acetylcytidine, ac4C	5.61	286.1034	0.2
2′-O-Methyladenosine, Am	5.64	282.1204	2.8
N6-methyladenosine, m6A	6.31	282.1206	3.2
N6,N6-dimethyladenosine, m6,6A	7.93	296.1354	0.2
N6-threonylcarbamoyladenosine, t6A	8.04	413.1417	0.8

^a^ ± 0.01 min

^b^Mass of protonated precursor ion

^c^Reference to the monoisotopic mass of protonated precursor ion

Before determining whether there were any variations on the level of each specific RNA modification, the use of our method to perform accurate quantitative analysis was evaluated. Specifically, a calibration experiment with a series of standard dilutions was performed. The results indicate the linearity and the dynamic range of the four canonical ribonucleoside standards match or exceed the earlier reports with < 6% relative standard deviation (n = 3) [19, 20].

**Fig. 1:**
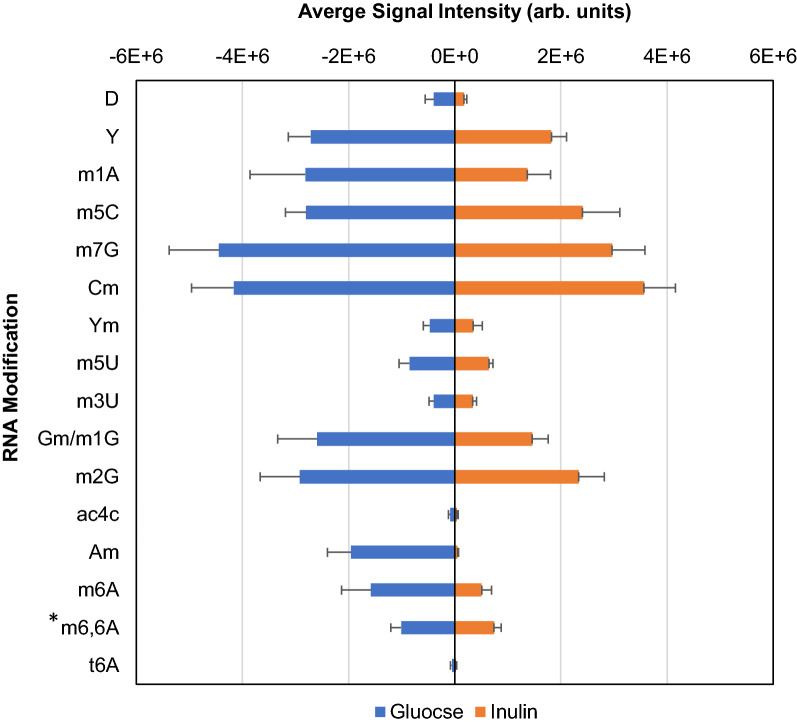
Mirrored histogram of glucose- or inulin-associated *L. agilis* epitranscriptomic profiles obtained from rRNA-depleted total RNA. *The signals of m6,6A were scaled down 10-folds. Each error bar represents one standard deviation with n ≤ 12

As shown by the error bars in Fig. 1, there was no significant variation on the level of each RNA modification among the four repeated profiling of glucose-associated *L. agilis* epitranscriptome. This indicates the *L. agilis* epitranscriptome reaches an equilibrium state when the cells were harvested during the exponential growth.

### Glucose-associated vs. inulin-associated *L. agilis* epitranscriptome

The reproducibility of the *L. agilis* epitranscriptomic profile prompted us to investigate whether the *L. agilis* epitranscriptome would become different when different prebiotic was used. As shown in Fig. 1, the RNA modifications found in the inulin-associated *L. agilis* epitranscriptome match with those listed in Table 1. However, there is an obvious downward trend when the cells were cultivated in inulin instead of glucose. However, the fold change of each individual RNA modification was not uniform, with 2′-O-methyladenosine (Am) to be down regulated most. From the chemical point of view, the 2′-O-methylation can disrupt the interactions between 2′-O-methylated RNA and RNase, thus protecting the 2′-O-methylated RNA from the RNase activity [21]. Therefore, when the level of Am was lowered in the inulin-associated *L. agilis* transcriptome, it would allow the *L. agilis* transcriptome to be turned over more effectively via the RNase digestion, which could be one way to rearrange the composition of the *L. agilis* transcriptome.

Among all seventeen RNA modifications witnessed in the *L. agilis* epitranscriptome, six of them were down regulated more than the average fold change of 0.65. The top six down-regulated RNA modifications include dihydrouridine (D), 1-methyladenosine (m1A), 4-acetylcytidine (ac4C), 2′-O-methyladenosine (Am), *N*6-methyladenosine (m6A) and *N*6-threonylcarbamoyl-adenosine (t6A). In the case of D modification, the hydrogenation at the 5 and 6 positions of uridine eliminate the only π bonding, thus weakening the effects of base stacking [22]. Whereas, the modifications of m1A, ac4C, m6A and t6A would interfere with the Watson–Crick base pairing. Therefore, the down regulation of those modifications could potentially change some of the RNA folding and/or annealing.

**Fig. 2:**
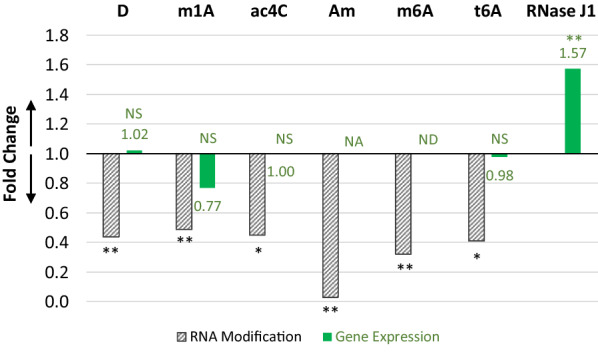
Fold change on the top six down-regulated RNA modifications and the expression level of their corresponding writer gene or RNase J1 that resulted from switching glucose to inulin in the cultivating *L. agilis*. The numerical numbers show the exact fold changes in the expression level. *NS *not significant, *NA *gene identity not available, *ND *transcript not detected; *p ≤ 0.05, **p ≤ 0.01

To investigate the underlying reason for the down regulation of *L. agilis* epitranscriptome, we performed an untargeted gene expression analysis and compared the expression levels in the cell cultures. First, we focused on the writer and eraser genes that correspond to the top six down-regulated RNA modifications (Fig. 2). However, the DNA sequence of those eraser genes are not known. Thus, our data analysis was limited to the writer genes. The results showed that there was no difference on the expression levels of the writer for t6A, D, m1A and ac4C modifications. The writer gene for Am in *L. agilis* is not known; and no transcript corresponding to the m6A writer gene could be detected. An alternative mechanism to lower the level of specific RNA modifications could be due to an increased cellular activity on degrading the modified RNA molecules. For this reason, the expression levels of all detectable ribonuclease in *L. agilis* were compared, which included RNase 3, RNase HI, RNase HII, J1, RNase R, RNase Y and RNase Z. In the case of RNase J1, a significant increase on its expression level was found among the inulin samples (Fig. 2). Whereas, no differences were detected for all the other RNases. Hence, based on the scope of this study, we speculate the down regulation of the inulin-associated *L. agilis* epitranscriptome could be linked to the higher expression level of RNase J1 when inulin was used instead of glucose to cultivate the cells.

## Conclusion

When *L. agilis* cells were cultivated with glucose being the sole source of energy, the *L. agilis* epitranscriptome consists of seventeen different RNA modifications at variable abundancy. There was a downward trend across the entire *L. agilis* epitranscriptome when the cells were exposed to inulin instead of glucose. To the best of our knowledge, this marks the first report on a system-wide variation of a bacterial epitranscriptome that resulted from adapting to an alternative source of energy. Based on our comparative study on gene expression, the down-regulated inulin-associated *L. agilis* epitranscriptome could be linked to an elevated RNase J1 activity. Overall, these results further strengthen the association of a unique epitranscriptome to a specific cellular activity [23].

## Limitations

Although all the above observations were limited to the selected strain of *L. agilis*, the experimental approach is applicable to study the other strains. For the MS analysis, due to the lack of available standards, the identification of each RNA modification was limited to the intrinsic accuracy from using high resolution mass spectrometry. Furthermore, the RNA modifications below the limit of detection would not be detectable and missed out from the reported profile.

## Data Availability

The datasets used and/or analyzed during the current study are available from the corresponding author on reasonable request.

## Abbreviations

ac4C: 4-Acetylcytidine
Am: 2′-O-Methyladenosine
D: Dihydrouridine
PCR: Polymerase chain reaction
*L. *agilis: *Lactobacillus agilis*
m1A: 1-Methyladenosine
m6A: *N*6-Methyladenosine
MS: Mass spectrometry
RNA: Ribonucleic acid
RNase: Ribonuclease
rRNA: Ribosomal ribonucleic acid
t6A: *N*6-Threonylcarbamoyl-adenosine
UPLC-MS/MS: Ultra-high performance liquid chromatography tandem mass spectrometry
